# Normal-Weight Obesity Is Associated with Increased Cardiometabolic Risk in Young Adults

**DOI:** 10.3390/nu12041106

**Published:** 2020-04-16

**Authors:** María Correa-Rodríguez, Katherine González-Ruíz, David Rincón-Pabón, Mikel Izquierdo, Antonio García-Hermoso, Cesar Agostinis-Sobrinho, Nohora Sánchez-Capacho, Marcela América Roa-Cubaque, Robinson Ramírez-Vélez

**Affiliations:** 1 Faculty of Health, Department of Nursing, University of Granada, Av. Ilustración, 60, 18016 Granada, Spain; macoro@ugr.es; 2 Grupo de Ejercicio Físico y Deportes, Vicerrectoría de Investigaciones, Universidad Manuela Beltrán, Bogotá DC 110231, Colombia; katherine.gonzalez@docentes.umb.edu.co; 3 ZIPATEFI (Zona de Investigaciones de Posgrados, Terapia Respiratoria y Fisioterapia de Areandina), Fundación Universitaria del Área Andina, Pereira 110231, Colombia; nicheunal@hotmail.com; 4 Department of Health Sciences, Public University of Navarra, Navarrabiomed- IdiSNA, Complejo Hospitalario de Navarra (CHN), 31008 Pamplona, Spain; mikel.izquierdo@gmail.com (M.I.); antonio.garcia.h@usach.cl (A.G.-H.); 5 CIBER of Frailty and Healthy Aging (CIBERFES), Instituto de Salud Carlos III, 28029 Madrid, Spain; 6 Laboratorio de Ciencias de la Actividad Física, el Deporte y la Salud, Facultad de Ciencias Médicas, Universidad de Santiago de Chile, USACH, Santiago 7500618, Chile; 7 Faculty of Health and Sciences, Klaipeda University, 92294 Klaipeda, Lithuania; cesaragostinis@hotmail.com; 8 Facultad de Ciencias de la Salud- Universidad de Boyacá, Tunja 150003, Colombia; nsanchez@uniboyaca.edu.co (N.S.-C.); maroa@uniboyaca.edu.co (M.A.R.-C.)

**Keywords:** body composition, normal-weight obesity, body fat, cardiometabolic risk, young adults

## Abstract

Normal-weight obesity (NWO) has been shown to be associated with cardiometabolic dysfunction. However, little is known regarding this potential relationship in early adulthood. The aim of this study was to investigate the associations between NWO and cardiometabolic risk factors in a large population of Colombian young adults. A cross-sectional study was conducted on 1354 subjects (61% women), aged from 18 to 30. Anthropometric data, including body mass index (BMI) and waist circumference (WC), were estimated, and the percentage of fat mass was measured through bioelectrical impedance analysis (BIA). Muscular fitness was determined by using a handgrip strength test and normalized grip strength (NGS = handgrip (kg)/body mass (kg)). A cardiometabolic risk Z-score was derived by assessing WC, triglycerides, high-density lipoprotein cholesterol (HDL-C) cholesterol, fasting glucose, and systolic blood pressure. NWO was defined by the combination of excess %BF (over 25.5% for men and 38.9% for women) and a BMI < 25 kg/m^2^. The overall prevalence of NWO was 29.1%. Subjects with NWO have an increased risk of cardiometabolic risk compared to the normal-weight lean group (OR = 3.10). Moreover, NWO was associated with an increased risk of presenting low HDL-C (OR = 2.34), high abdominal obesity (OR = 7.27), and low NGS (OR = 3.30), *p* < 0.001. There is a high prevalence of NWO in American Latin young adults and this condition is associated with an increased cardiovascular risk, high blood pressure, low HDL-C, high abdominal obesity, and low muscular strength early in life. Screening for adiposity in subjects with a normal BMI could help to identify young adults at a high risk of cardiometabolic abnormalities.

## 1. Introduction

Excessive body fat increases the risk of other non-communicable diseases (NCD), such as cardiovascular disease (CVD), chronic respiratory disease, metabolic disorders, and certain types of malignant neoplasms [1,2]. Disturbances in adipose tissue function may induce alterations in adipose tissue metabolism (i.e., lipid metabolism/lipolysis) and the storage capacity of dietary lipid in adipose tissue. As a consequence, the body is not effectively able to adapt to its metabolic or energy demands, also described as metabolic inflexibility [2]. Although different etiologies precede the development of NCD, body fat accumulation is a major contributing risk factor, and is therefore a relevant target of scientific research [3].

The World Health Organization (WHO) defines obesity as excessive body fat accumulation, which is associated with several risks to health [1]. The body mass index (BMI), is used as a surrogate marker for body fat and for classifying obesity, is positively associated with risk factors for cardiovascular and metabolic diseases when BMI is above 18.5 kg/m^2^ [4]. However, BMI is not a very accurate measurement for estimate adiposity in a particular individual since BMI does not take body composition (i.e., fat-free mass vs. adipose tissue) into account. Thus, an individual with healthy BMI (18.5–24.9 kg/m^2^) may have either an appropriate body fat percentage or an excessive body fat accumulation that might be masked by the normal BMI [5]. 

In this context, excessive body fat, despite healthy body weight, has been described as normal-weight obesity syndrome (NWO) [6]. NWO is a condition in which individuals who have normal body weight and BMI but high body fat percentage, are at a greater risk of developing NCD [6,7,8]. In the US population, estimates show that about 30 million Americans are affected by NWO [8]. Previous studies have reported associations between NWO and metabolic disorders [9]. Similarly, individuals aged >20 years with NWO were four-fold more likely to develop metabolic syndrome (MetSyn) than those with normal BMI and healthy body fat percentage (16.6% vs. 4.8%) [8]. In females of Caucasian origin aged 35–75 years from Switzerland, women with NWO had a higher cardiometabolic risk and higher prevalences of high waist circumference (WC), high triglycerides, low high-density lipoprotein cholesterol (HDL-C), and hyperglycaemia but a similar prevalence of hypertension compared to lean women [10]. In addition, other studies have reported associations between NWO with the development of dyslipidemia [11], insulin resistance [12], and changes in blood pressure [13] as well as pro-oxidative effects and the exacerbation of low-grade chronic proinflammatory status in middle-aged adults [14]. Interestingly, a 7-year longitudinal study concluded that children and adolescents with NWO have an increased risk for cardiometabolic morbidity in adulthood [15].

Thus far, only one study about NWO in Latin America has been published [16]. It included 1222 young adults aged 23 to 25 years with normal BMI, 55.3% of whom were women. The prevalence of NWO (defined by the combination of excess body fat—the sum of triceps and subscapular skinfolds >P90 of the study sample—and normal BMI) among the total sample was 9.1% (9.2% for men and 9.0% for women). In this study, these authors also observed an association between NWO, low levels of HDL-C (OR = 1.65), high triglycerides (OR = 1.93), and high WC (OR = 8.46). 

Considering that body composition is not routinely assessed in outpatient care, it is important to characterize NWO syndrome and to identify the health risks associated with this condition. To our knowledge, there are few studies reporting an association between NWO and metabolic disorders exclusively in young adults or coming from Latin American countries [16]. Therefore, the aim of the present study was to evaluate the association between NWO and cardiometabolic risk factors in young adults from Colombia.

## 2. Materials and Methods

### 2.1. Design and Study Population

This study was a secondary analysis of data from the FUPRECOL study, which is a non-representative survey conducted between 2014 and 2017 on 1842 collegiate students (716 men and 1126 women) from Colombia. The design of the study and data collection has previously been described in detail [17]. For this analysis, we used data from 1354 participants included as a subsample with NWO-components. The participants answered a questionnaire (paper-and-pencil format) containing information on socioeconomic, demographic, and behavioural variables (alcohol intake, physical activity (PA) “proxy”, tobacco), and underwent physical examination when they were 18–30 years of age. Participants who had a clinical diagnosis of a major systemic disease, including conditions such as cancer, systemic lupus erythematosus, diabetes mellitus, chronic inflammatory conditions, such as rheumatoid arthritis, hypothyroidism or hyperthyroidism, multiple sclerosis, and infectious conditions, were excluded from the analyses. Signed informed consent was obtained from all FUPRECOL study participants. The protocol was in accordance with the Declaration of Helsinki (World Medical Association for Human Subjects) and its later amendments. All procedures have been approved by the ethics committee of the UMB (Code N° 01-1802-2013).

### 2.2. Anthropometric Measurements 

All physical examinations were carried out by Level 2 experts certified by the International Society for the Advancement of Kinanthropometry (ISAK) in accordance with ISAK guidelines [18]. Weight was measured to the nearest 100 g using a scale (Model Tanita^®^ BC-420^®^, Tokyo, Japan). Height was measured to the nearest 0.1 cm using a stadiometer (Seca^®^ 274, Hamburg, Germany). BMI was calculated as body mass (kg)/height (m^2^). WC (abdominal obesity) was measured at the waist (at the midpoint between the last rib and the iliac crest) after gentle expiration with a non-elastic flexible tape measure (Lufkin W606PM^®^, Parsippany, NJ, USA), as recommended by the ISAK guidelines [18]. Waist-to-height ratio (WtHR) was calculated as the ratio of WC to height, with obesity defined as >0.49 for men and >0.50 for women according to previous reports [4]. NWO was defined as a BMI < 25 kg/m^2^ and a % body fat (%BF) over the gender-specific in collegiate students (25.5% for men and 38.9% for women) [19].

The percentage of body fat (%), the visceral fat score/levels, and fat free mass (kg), were determined by bioelectrical impedance analysis (BIA) (Tanita BC 420 MA/SC-331S^®^, Tokyo, Japan). The BIA Monitor (Model BC 420 MA/SC-331S^®^) provides a visceral fat rating from 1–59, as previously described [20]. The fat mass index (FMI) was calculated by dividing each subject’s fat mass (kg) by the square of his/her height (m), as previously described [21]. Then, four variables (the ratio of fat mass (kg) to fat free mass (kg), the ratio of fat mass (kg) to handgrip strength (kg), and the ratio of handgrip strength (kg) to fat free mass (kg)), normalized as handgrip strength to visceral fat level, were calculated [17,19,21].

### 2.3. Clinical Measurements

Blood samples were drawn in the morning after 10–12 h of fasting by a trained technician, between 07:00 and 09:00 a.m. Capillary blood samples (40 µL) were collected to determine biochemical parameters, including of fasting glucose, HDL-C, triglycerides (TG), and total cholesterol (TC), using Cardiocheck^®^ equipment (Mexglobal SA, Parsippany, NJ, USA). LDL-C was measured using Friedewald’s Formula LDL-C = Total Cholesterol − HDL-C − TG/5 if triglyceride values were ≤ 400 mg/dL [22].

For the blood pressure (BP) measurements, we used an Omron^®^ digital sphygmomanometer model HEM 705 CP (Omron^®^ Healthcare Europe B.V., Hoofddorp, Netherlands), with the participants seated, following the recommendations of the European Heart Society [23], after 5 min rest. The mean arterial blood pressure (MAP) was calculated as MAP = diastolic BP + (0.333 × (systolic BP × 2 diastolic BP)).

A cardiometabolic risk Z-score (CMR) was created from the sum of systolic (BP), TG, WC, HDL-C, and fasting glucose Z-score [24]. This cluster was constructed as the sum of the Z-scores of each variable, which was calculated as follows: (value − mean)/standard deviation (SD), separately for men and women, and for each 1-year age group. Individuals with a cardiometabolic risk Z-score + 1 SD above the mean were identified as having increased cardiometabolic risk, with a lower cardiometabolic risk Z-score being indicative of a healthier risk profile.

Grip strength was tested by a digital dynamometer (T.K.K. 5401, Grip-D Smedley, Takei, Japan), with adjustment for the hand size of each participants. The best score for each hand was recorded in kilograms (kg), accurate to one decimal place, and the score (kg) was calculated as the average of the scores for the left and right hands [20]. The grip strength was normalized as handgrip strength (NGS) per body mass, i.e., (handgrip strength in kg)/(body mass in kg). 

To assess the degree of adherence to the Mediterranean diet (MedDiet), the validated 14-item Mediterranean Diet Adherence questionnaire was administered [25]. This tool was developed in the PREDIMED study [26]. This questionnaire comprises 12 items about food consumption frequency and two on the consumption habits of foods considered characteristic of the Med Diet pattern. Each question is scored either 0 or 1 and the total score ranges from 0 to 14; the higher the score, the greater the adherence to the Med Diet.

### 2.4. Statistical Analysis

The sample characteristics were presented as the mean and standard deviation (SD) for continuous variables, and frequencies and percentages were calculated for categorical variables. Both statistical (Kolmogorov–Smirnov test) and graphical methods (normal probability plots) were used to examine the fit to a normal distribution for each continuous variable. Due to their skewed distribution, all dependent variables were log-transformed before to be included in the models. To aid interpretation, the data were back-transformed from the log scale for presentation in the results (Table 1). 

Analysis of covariance (ANCOVA) was used to compare means to explore group differences (i.e., NWL vs. NWO), adjusted for age and sex. Finally, the odds ratios (ORs) and 95% confidence interval (CI) for cardiometabolic risk factors were calculated in each group using a multiple logistic regression analysis after adjusting for age and sex (Model 1). The analysis was further adjusted for physical activity, alcohol intake, tobacco status, and healthy diet (Model 2). All p-values presented are two-tailed, and *p*-values of <0.05 were considered to indicate statistical significance. Statistical analyses were performed with SPSS ver. 24.0 for Windows (SPSS, Inc., Chicago, IL, USA).

## 3. Results

The number of normal-weight subjects included in the study was 1354 (61% females). Most anthropometric, physical, and cardiometabolic characteristics differed significantly between women and men (all *p* < 0.05), except for age (*p* = 0.215), triglycerides (*p* = 0.172), and cardiometabolic risk Z-score (*p* = 0.834). Low HDL-cholesterol was significantly higher in women (67.5%) than in men (50.1%), whereas high blood pressure was higher in men (35.0%) compared with women (12.4%). High LDL-cholesterol was also higher in men (27.0%) than in women (18.2%). High WtHR, WC, fat mass index, the ratio of fat mass (kg) to fat free mass (kg), the ratio of fat mass (kg) to handgrip strength (kg), the ratio of handgrip strength (kg) to fat free mass (kg), the NGS to visceral fat level ratio, glycemia, triglycerides, cardiometabolic risk Z-score (+1 SD above the mean), alcohol intake, or smoking did not differ by sex. 

For the purpose of the present study, the analyses were restricted to the NWL and NWO groups. In the sample, 29.1% had NWO (46.0% women). Regarding anthropometrics parameters, the NWO group had significantly higher values for body weight, height, WC, fat free mass, visceral fat level, fat mass index, the ratio of fat mass (kg) to fat free mass (kg), the ratio of fat mass (kg) to handgrip strength (kg), the ratio of handgrip strength (kg) to fat free mass (kg), and the NGS to visceral fat level ratio (all *p* < 0.001), than the NWL group. In addition, systolic BP, diastolic BP, MAP, total cholesterol, LDL-C, TG, fasting plasma glucose, and cardiometabolic risk Z-score were significantly greater in NWO compared to those in the NWL group, and smaller in HDL-C (all *p* < 0.05). Finally, the NWO group was weaker compared to the NWL group (7.2% versus 15.8%, *p* < 0.05).

**Figure 1:**
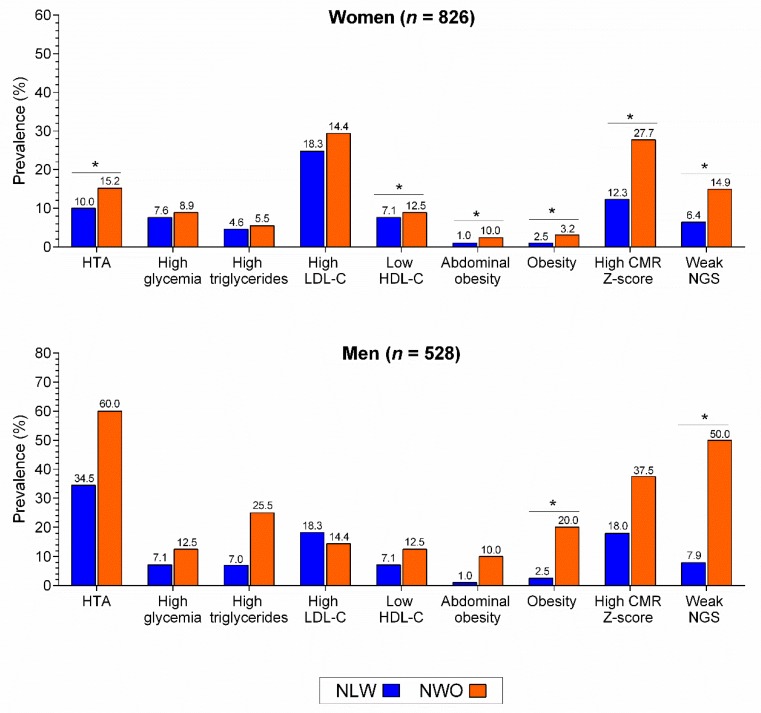
Differences in cardiometabolic risk parameters muscular fitness between NLW and NWO. HTA, hypertension; LDL-C, Low-density lipoprotein cholesterol; HDL-C, High-density lipoprotein cholesterol; CMR, cardiometabolic risk; NGS, normalized as handgrip strength. * *p* < 0.01.

High blood pressure, low HDL-cholesterol, abdominal obesity, obesity by high WtHR, high cardiometabolic risk Z-score, and weak NGS were significantly higher in NWO compared to NLW in women (all *p* < 0.05), whereas obesity and weak NGS only in men (*p* < 0.05), Figure 1.

**Figure 2:**
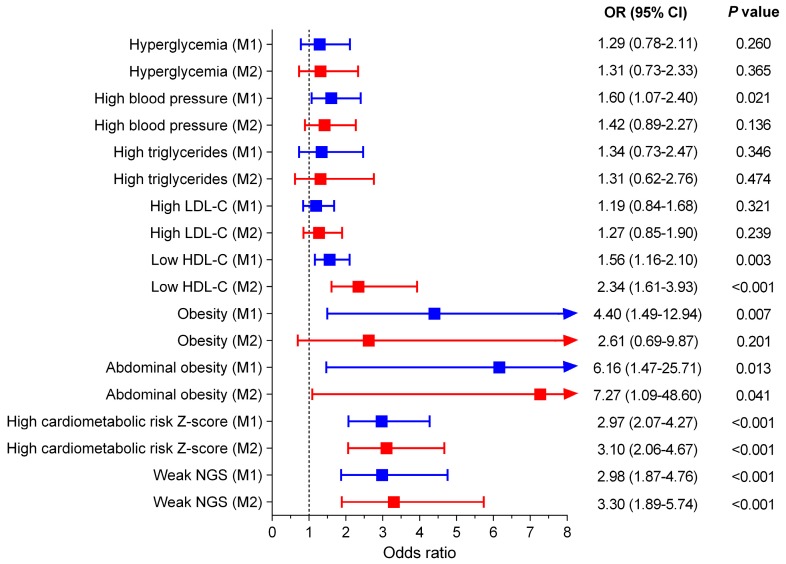
Multiple logistic regression analysis for having cardiometabolic risk factors in the NWO group. The adjusted odds ratios (ORs) for having cardiometabolic risk factors in the NWO group were investigated using a multiple logistic regression analysis and compared to those of the NWL group (Ref). HDL-C, High-density lipoprotein cholesterol; LDL-C, Low-density lipoprotein cholesterol; NGS, normalized as handgrip strength; Model 1 (M1) adjusted for age and sex; Model 2 (M2) adjusted for the same covariates as Model 1 and physical activity “proxy”, alcohol intake, smoking, and adherence Mediterranean diet.

The adjusted ORs for having cardiometabolic risk factors in the NWO group were investigated using a multiple logistic regression analysis and compared to those of the NWL group (Figure 2). Age and sex in Model 1 (M1), in addition to smoking status, alcohol intake, physical activity “proxy”, and adherence to the Mediterranean diet in Model 2 (M2), were adjusted in the multiple logistic regression analysis. After adjustment for age and sex (Model 1), high blood pressure (OR = 1.60, 95%CI 1.07–2.40), low HDL-C (OR = 1.56, 95%CI 1.16–2.10), abdominal obesity (OR = 6.16, 95%CI 1.47–25.71), obesity for high WtHR (OR = 4.40, 95%CI 1.49–12.94), high cardiometabolic risk Z-score (OR = 2.97, 95%CI 2.07–4.27), and weak NGS (OR = 2.98, 95%CI 1.87–4.76) were the potential factors with the highest values in the risk of occurrence of the NWO group (*p* < 0.05). In addition, the results from multiple logistic regression analyses in Model 2 were also consistent except for high blood pressure (OR = 1.42, 95%CI 0.89–2.27), and obesity for high WtHR (OR = 2.61, 95%CI 0.69–9.87), Model 2.

## 4. Discussion

The presence of cardiometabolic risk factors in early ages has been associated with the earlier onset of chronic conditions, including diabetes, heart disease, and risk of early mortality [27]. Thus, to identify whether the condition of NWO is linked to a cardiometabolic risk profile in young adults is especially relevant. This study reported that NWO was associated with an increased cardiovascular risk, high blood pressure, low HDL-C, high abdominal obesity, and low muscular strength in a large cohort of young adults from Colombia, supporting that there is a high prevalence of clustering of cardiometabolic abnormalities among subjects with NWO. Due to the deleterious effect of NWO on cardiometabolic profile, adiposity should be routinely assessed in clinical practice in order to identify young adults with an NWO condition. Failure to recognized NWO in early adulthood may contribute to the lack of prescription to adopt healthy lifestyle changes that might prevent future cardiometabolic disturbances later in life. 

In this study, differences in the obesity groups according to age (effect size = 0.325), height (effect size = 0.607), and BMI (effect size = 0.095) were identified. Although both parameters are important factors associated with fatness among young people, it should be noted that these differences have a relatively small to moderate clinical significance that may be due to the non-probability sampling design study. Despite this, all comparisons were adjusted for age and sex (M1). 

In our study, the overall prevalence of NWO, defined by the combination of excess %BF (over 25.5% for men and 38.9% for women) and a BMI < 25 kg/m^2^, was 29.1%. Note that that 2.0% of the men and 46% of the women students were classified as NWO. These findings evidenced the important prevalence of NWO during early adulthood in a Latin American population. Among the same line, Kim et al. [28] reported a prevalence of 32% of NWO in Korean adults aged 20 years or older and Romero-Corral et al. [8]. found an overall prevalence of 33.4% in Americans. These results differ from the results from Madeira et al. [16], who reported that the prevalence of NWO among Brazilian young adults aged 23–25 years was 9.1%. However, it should be noted that, in this study, body fat was estimated by measuring skinfold thickness and the cutoff point for NWO was the 90th percentile of the sum of the subscapular and triceps skinfolds. Similarly, using the highest tertile of body fat percentage as the cutoff value, in a study conducted in adults aged 35–75 years from Switzerland, the prevalence of NWO was 5.4% in women and less than 3% in men [14]. Therefore, the discrepancies in the NWO prevalence data might be due to the lack of consensus regarding diagnostic criteria, characteristics of the participants (age, lifestyle, dietary habits), and ethnic differences in study cohorts [9]. 

**Figure 3:**
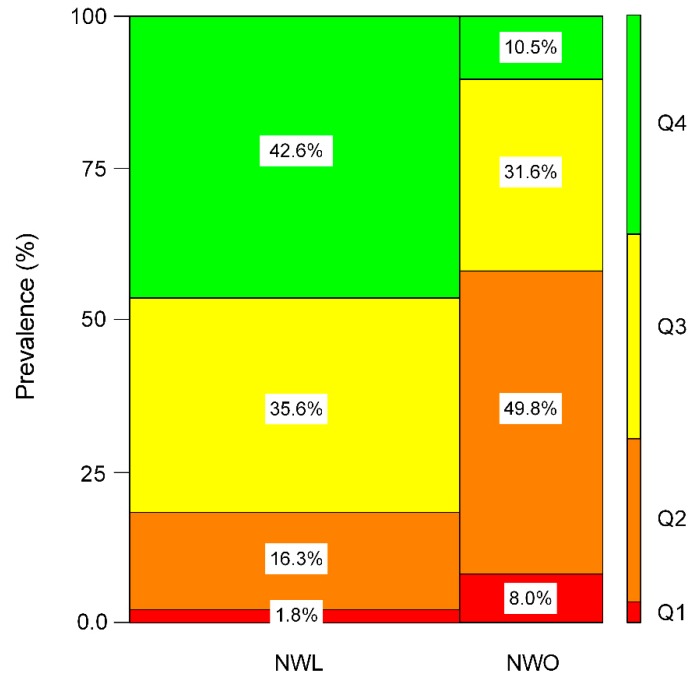
Mosaic plot depicting the frequency of participants (%) in each obesity group (NWL, NWO) and quartiles (Q1 to Q4) to NGS and the visceral fat level ratio. Normal-weight lean (NWL) and normal-weight obesity (NWO). Q1 (lower or “unhealthy” group), p < 0.001. The NGS to visceral fat level ratio was divided into quartiles with the following (min–max) values: Q1: 0.01–0.13, Q2: ≥0.13–0.33, Q3: ≥0.33–0.49 and Q4: >0.49–0.99.

In agreement with previous findings, the current study supports the notion that young adults with a normal BMI but excessive fatness perform worse on the six measures of fatness (WC, WtHR, fat free mass, fat mass index, visceral fat level, and fat mass to fat free mass ratio), muscular fitness parameters, and combined fitness vs. fatness index (ratio of fat mass to handgrip strength, ratio of handgrip strength to fat free mass, and ratio of NGS to visceral fat level) [29]. To illustrate the degree to which both concepts overlap, we reported the prevalence of each obesity group according to the NGS to visceral fat level ratio quartiles (first quartile (Q1 lowest group, “unhealthy”), second quartile (Q2), third quartile, (Q3) and fourth quartile (Q4 highest group, “healthy”), Figure 3. The category containing the NWL group was 1.8% in Q1, 16.3% in Q2, 35.6% in Q3, and 42.6% in Q4. In addition, the proportions of subjects with NWO were 8.0% in Q1, 49.8% in Q2, 31.6% in Q3, and 10.5% in Q4. These findings indicate that those subjects positioned in Q4 and the NWL group present better body composition than the NWO group (42.6% vs. 10.5%), within normal BMI values, *p* < 0.001.

An increase in body composition markers is considered a risk factor for cardiovascular disease in young adults [30,31] and, in this study, after adjusting for a potential confounding factors, the NWO group had higher ORs for “proxy” indicators of adiposity (WtHR) than the lean group. Our results are in line with the previous study that reported an association between increased abdominal adiposity and WHtR and cardiovascular disease and cardiovascular risk factors even among subjects with a normal BMI [32,33,34]. In our present study, the participants in Q1 to Q2 showed the lowest prevalence of NGS to visceral fat level ratio (18.1%) in the NWL group, whereas the NWO group showed a higher prevalence of NGS to visceral fat level ratio (57.8%), *p* < 0.001. In agreement with these findings, previous studies conducted on young people have reported an inverse association between muscle mass, body composition markers and/or metabolic profile, and cardiovascular risk factors [35,36,37]. Therefore, the body composition is an essential tool for estimating cardiometabolic risk and identifying young adults within normal BMI values.

Considering the interaction of reduced skeletal muscle mass/muscular strength with elevated fatness markers, we used the three surrogate index (ratio of fat mass to fat free mass, ratio of fat mass to handgrip strength, and ratio of handgrip strength to fat free mass) to investigate its relationship with NWO syndrome. For example, we found that young adults with NWO showed significantly lower handgrip strength (in terms of relative and absolute values) to fat free mass compared to subjects with NWL, with healthy BMI values. In line with our results, reduced skeletal muscle mass and visceral fat area were reported to increase the risk of metabolic impairment more than any other single factor alone in Korean adults with metabolic syndrome and type 2 diabetes [38] or college students in Colombia [35]. Similarly, in a longitudinal study (7 years), Wiklund et al. [15] found that NWO girls (from age 11 to 18) had a greater amount of body fat and a stable lean mass/fat mass ratio index from childhood to adulthood compared to their healthy weight and healthy BMI peers. Taken together, the results suggest that it would be of interest that clinical physicians identify subjects with a high risk of NWO.

NWO is a state in which an excessive amount of fatness markers and decreased lean mass is accompanied by average/normal BMI values. In this line, we demonstrated that the excess of body fat in subjects with NWO has an increased risk of cardiometabolic risk Z-score compared to NWL (OR = 3.10). Moreover, NWO was associated with an increased risk of presenting low HDL-C (OR = 2.34), high abdominal obesity (OR = 7.27), and low muscular strength (OR = 3.30). These findings are in agreement with a previous study conducted in young adults from a middle-income country that found associations between components of the metabolic syndrome, including high waist circumference and low HDL-C early in life [16]. Marques-Vidal also concluded that swiss women aged 35–75 with NWO present higher cardiovascular risk factors than lean women [14] and Moy et al. [39] indicated that women with NWO had cardiometabolic abnormalities, including abdominal obesity, dyslipidemia, and increased blood pressure. Another study carried out using data from the Third National Health and Nutrition Examination Survey (NHANES III) in adults > 20 years demonstrated that subjects with NWO also have a higher prevalence of dyslipidemia, hypertension, and cardiovascular disease [8], and Shea et al. [11] Interestingly, a large study that estimated %BF with air displacement plethysmography showed that non-obese individuals according to BMI but obese based on body fat have higher values of WC, blood pressure, triglycerides, glucose, insulin, HOMA, and lower values of HDL-C [40]. Indeed, for the first time, we found that NWO is also associated with low muscular fitness in early adulthood. In this line, a recent study reported that NWO is associated with poorer physical fitness and the relationship is partially mediated by lower skeletal muscle mass in Chinese university students [29]. It should be highlighted that, similar to the study of Madeira et al. [16], no association was observed in our study between NWO and high blood pressure in the fully adjusted model. Note that this study and our research were conducted only in cohorts of young adults, whereas the above-mentioned studies were carried out in populations with wide age ranges. Thus, differences in risk estimates between NWO and cardiometabolic disturbances might be explained by differences in the age and ethnicity of the study populations and the lack of consensus regarding the NWO definition. 

Supporting our observations, previous studies have also shown that only measuring BMI might not be sufficient criteria for identifying individuals at cardiometabolic risk since it might fail to identify subjects who, despite having a normal BMI, present an excess of adiposity and are also at a high risk of cardiometabolic imbalances and, consequently, cardiovascular diseases. This study provides important insights into understanding obesity since it states that a normal BMI might not necessarily imply cardiometabolic protection. The screening of NWO might be useful for clinicals in order to implement effective strategies to prevent cardiometabolic diseases. In addition, taking into account that NWO is associated with an increased risk of cardiometabolic risk, there is a need to establish an appropriate criterion to define NWO. Our research might also encourage authors to reach a consensus regarding the diagnostic criteria for NWO. Interestingly, since recent studies indicated that oral condition might affected metabolic risk [41,42], it should of interest to examine the potential relationship between NWO and oral health.

This study has potential limitations that should be addressed. First, the cross-sectional design does not allow us to explain causality. Second, the prevalence of NWO was low (39.1% in the overall population, 46.0% in women, and 2.0% in men), which might limit the generalizability of our results. However, this prevalence is similar to that reported in other epidemiological studies [14]. Thus, future prospective analysis is necessary to determine any relationship between the NWO condition and cardiometabolic risk factors. Third, our study comprised a non-representative sample of young adults from Colombia, making the generalizability of the results to populations with different characteristics difficult. Last, the study population included only university students. However, this also might be considered as a strength since it eliminates the potential confounding effect of age. Despite these limitations, the main strength of our study is that, to our knowledge, this is the largest research on the relationship between NWO and cardiometabolic risk in a population of Latin American young adults. Furthermore, highly standardized procedures were developed within the FUPRECOL study to avoid measurement bias. Additionally, statistical models were adjusted for several variables, including age, sex, smoking, alcohol intake, physical activity “proxy”, or healthy diet. 

## 5. Conclusions

In conclusion, there is a high prevalence of NWO in American Latin young adults, and this condition is associated with an increased cardiovascular risk, high blood pressure, low HDL-C, high abdominal obesity, and low muscular strength early in life. Our results suggest that screening for adiposity in subjects with a normal BMI could help to identify young adults at a high risk of cardiometabolic abnormalities. 

## Figures and Tables

**Table 1:** Characteristics of the study sample of normal-weight lean (NWL) and normal-weight obesity (NWO) participants.

Characteristics	Women (*n* = 826)	Men (*n* = 528)	NWL (*n* = 961)	NWO (*n* = 393)
Age (years)	20.4 ± 2.2	20.2 ± 2.1	20.1 ± 2.1	20.8 ± 2.2 **
**Anthropometric parameters**				
Body weight (kg)	54.4 ± 6.2	64.1 ± 7.9 **	57.7 ± 9.4	59.1 ± 4.9 **
Height (cm)	159.2 ± 5.8	172.2 ± 6.5 **	165.6 ± 9.4	160.9 ± 5.6 **
Body mass index (kg/m^2^)	21.4 ± 2.1	21.6 ± 2.1 **	20.9 ± 1.5	22.8 ± 1.5 **
Waist circumference (cm)	68.3 ± 5.4	74.6 ± 5.9 **	70.3 ± 6.9	71.9 ± 4.6 **
WtHR	0.43 ± 0.04	0.43 ± 0.03 *	0.42 ± 0.03	0.44 ± 0.03 **
Fat free mass (kg) ^a^	41.0 ± 3.0	55.5 ± 5.9 **	47.8 ± 9.1	42.3 ± 2.3 **
Visceral fat level	1.3 ± 0.9	1.5 ± 1.1 **	1.3 ± 0.9	1.7 ± 1.0 **
Fat mass index (kg/m^2^)	6.50 ± 2.75	3.81 ± 2.24 **	3.39 ± 1.23	6.56 ± 0.85 **
Ratio of fat mass (kg) to fat free mass (kg)	0.38 ± 0.14	0.18 ± 0.10 **	0.19 ± 0.08	0.39 ± 0.04 **
Ratio of fat mass (kg) to handgrip strength (kg)	0.69 ± 0.31	0.29 ± 0.18 **	0.32 ± 0.16	0.71 ± 0.16 **
Ratio of handgrip strength (kg) to fat free mass (kg)	0.57 ± 0.12	0.68 ± 0.12 **	0.63 ± 0.14	0.57 ± 0.10 **
Ratio of NGS to visceral fat level	0.30 ± 0.17	0.38 ± 0.25 **	0.47 ± 0.16	0.29 ± 0.13 **
**Muscular fitness**				
Handgrip strength (kg)	23.8 ± 4.6	38.8 ± 7.1 **	31.6 ± 9.8	24.7 ± 4.9 **
NGS (kg/kg)	0.44 ± 0.08	0.61 ± 0.11 **	0.54 ± 0.12	0.42 ± 0.08 **
Weak NGS, ≤ 0.47 kg in men and ≤ 0.33 kg in women, %	10.3	8.7	7.2	15.8 *
**Cardiometabolic parameters**				
Systolic blood pressure (mmHg)	109.9 ± 11.0	118.2 ± 12.3 **	112.1 ± 12.7	115.5 ± 10.8 **
Diastolic blood pressure (mmHg)	70.9 ± 8.2	72.5 ± 10.0 *	71.1 ± 9.3	72.5 ± 8.2 *
Mean arterial pressure (mmHg)	90.4 ± 8.7	95.5 ± 10.5 **	91.7 ± 10.2	94.0 ± 8.4 **
Total cholesterol (mg/dL)	145.5 ± 34.1	131.0 ± 28.9 **	137.6 ± 32.5	145.3 ± 33.2
LDL-C (mg/dL)	87.0 ± 26.2	80.2 ± 25.8 **	82.9 ± 25.1	87.9 ± 28.3
HDL-C (mg/dL)	45.5 ± 12.7	40.8 ± 10.4 **	44.5 ± 12.0	41.8 ± 12.2 *
Triglycerides (mg/dL)	82.7 ± 40.9	85.8 ± 39.0	83.4 ± 39.8	85.1 ± 41.0
Fasting plasma glucose (mg/dL)	85.3 ± 12.0	83.5 ± 11.8 *	84.4 ± 12.0	85.2 ± 11.6
Cardiometabolic risk Z-score	−0.85 ± 2.25	−0.87 ± 2.30	−1.17 ± 2.24	−0.09 ± 2.16 **
**Increased cardiometabolic risk, %**				
HTA, ≥130 mm Hg SBP and/ or DBP 85 mm Hg	12.4	35.0 **	16.4	23.2 *
Hyperglycemia, ≥5.6 mmol/L [100 mg/dL]	8.2	7.1	7.3	9.0
High triglycerides, ≥1.7 mmol/L [151 mg/dL]	5.0	7.3	5.9	6.0
High LDL-C, ≥2.6 mmol/L [100 mg/dL]	27.0	18.2 *	21.4	29.1
Low HDL-C < 1 mmol/L [38.7 mg/dL] in men and 1.3 mmol/L [50.3 mg/dL] in women	67.5	50.1 **	56.3	71.6 *
Obesity, WC ≥ 90 cm in men, and ≥ 80 cm in women	1.3	1.0	0.6	2.6 *
Obesity, WtHR > 0.49 in men and > 0.50 in women	1.8	2.9	1.7	3.6 *
Cardiometabolic risk Z-score, + 1 SD above the mean	19.5	18.3	15.4	27.9 *
**Self-report lifestyles, %**				
Adherence Mediterranean Diet	14.8	10.1 *	12.8	12.3
Alcohol (≥1 times/week)	5.4	7.6	6.1	9.0
Tobacco (≥10 cigarettes/week)	26.6	29.2	23.5	29.9
Physical activity “proxy” ≥ 150 min/week	29.7	37.2 *	35.3	24.9 *

The results are shown as mean and standard deviation (SD) for continuous variables, and percentages (%) for categorical variables. HDL-C, High-density lipoprotein cholesterol; LDL-C, Low-density lipoprotein cholesterol; NGS, normalized as handgrip strength. WC, waist circumference; WtHR, Waist-to-height ratio; HTA, hypertension; SBP, systolic blood pressure; DBP, diastolic blood pressure. Physical activity was assessed as an accumulated time of 150 min/week or more, undertaking moderate- to vigorous-intensity physical activity, which was considered to meet physical activity recommendations for adults. Alcohol consumption and smoking status were defined as subjects who had consumed any alcoholic beverage ≥1 times/week, and those who had smoked ≥10 cigarettes/week, for at least 6 months, as previously described by Ramírez-Vélez et al. (19). Participants who exercised five times a week for >30 min were categorised as ‘physically active’. Analysis adjusted for age and sex. ^a^ Fat free mass is comprised of muscle, bone, tissue, water, and all other fat free mass in the body; * *p* < 0.05; ** *p* < 0.001.

## References

[1] WHO (2015). Global Status Report on Noncommunicable Diseases 2010. https://www.who.int/nmh/publications/ncd_report2010/en/.

[2] Goodpaster B.H., Sparks L.M. (2017). Metabolic flexibility in health and disease. Cell Metab..

[3] Hocking S., Samocha-Bonet D., Milner K.L., Greenfield J.R., Chisholm D.J. (2013). Adiposity and insulin resistance in humans: The role of the different tissue and cellular lipid depots. Endocr. Rev..

[4] Di Angelantonio E., Bhupathiraju S.N., Wormser D., Gao P., Kaptoge S., de Gonzalez A.B., Cairns B.J., Huxley R., Jackson C.L., Joshy G. (2016). Body-mass index and all-cause mortality: Individual-participant-data meta-analysis of 239 prospective studies in four continents. Lancet.

[5] De Lorenzo A., Del Gobbo V., Premrov M.G., Bigioni M., Galvano F., Di Renzo L. (2007). Normal-weight obese syndrome: Early inflammation?. Am. J. Clin. Nutr..

[6] De Lorenzo A., Martinoli R., Vaia F., Di Renzo L. (2006). Normal weight obese (NWO) women: An evaluation of a candidate new syndrome. Nutr. Metab. Cardiovasc. Dis..

[7] Marques-Vidal P., Pécoud A., Hayoz D., Paccaud F., Mooser V., Waeber G., Vollenweider P. (2008). Prevalence of normal weight obesity in Switzerland: Effect of various definitions. Eur. J. Nutr..

[8] Romero-Corral A., Somers V.K., Sierra-Johnson J., Korenfeld Y., Boarin S., Korinek J., Jensen M.D., Parati G., Lopez-Jimenez F. (2010). Normal weight obesity: A risk factor for cardiometabolic dysregulation and cardiovascular mortality. Eur. Heart J..

[9] Franco L.P., Morais C.C., Cominetti C. (2016). Normal-weight obesity syndrome: Diagnosis, prevalence, and clinical implications. Nutr. Rev..

[10] Di Renzo L., Galvano F., Orlandi C., Bianchi A., Di Giacomo C., La Fauci L., Acquaviva R., De Lorenzo A. (2010). Oxidative stress in normal-weight obese syndrome. Obesity.

[11] Shea J.L., King M.T.C., Yi Y., Gulliver W., Sun G. (2012). Body fat percentage is associated with cardiometabolic dysregulation in BMI-defined normal weight subjects. Nutr. Metab. Cardiovasc. Dis..

[12] Kim J.Y., Han S.H., Yang B.M. (2013). Implication of high-body-fat percentage on cardiometabolic risk in middle-aged, healthy, normal-weight adults. Obesity.

[13] Kang S., Kyung C., Park J.S., Kim S., Lee S.P., Kim M.K., Kim H.K., Kim K.R., Jeon T.J., Ahn C.W. (2014). Subclinical vascular inflammation in subjects with normal weight obesity and its association with body Fat: An 18 F-FDG-PET/CT study. Cardiovasc. Diabetol..

[14] Marques-Vidal P., Pécoud A., Hayoz D., Paccaud F., Mooser V., Waeber G., Vollenweider P. (2010). Normal weight obesity: Relationship with lipids, glycaemic status, liver enzymes and inflammation. Nutr. Metab. Cardiovasc. Dis..

[15] Wiklund P., Törmäkangas T., Shi Y., Wu N., Vainionpää A., Alen M., Cheng S. (2017). Normal-weight obesity and cardiometabolic risk: A 7-year longitudinal study in girls from prepuberty to early adulthood. Obesity.

[16] Madeira F.B., Silva A.A., Veloso H.F., Goldani M.Z., Kac G., Cardoso V.C., Bettiol H., Barbieri M.A. (2013). Normal weight obesity is associated with metabolic syndrome and insulin resistance in young adults from a middle-income country. PLoS ONE.

[17] Garcia-Hermoso A., Tordecilla-Sanders A., Correa-Bautista J.E., Peterson M.D., Izquierdo M., Prieto-Benavides D., Sandoval-Cuellar C., González-Ruíz K., Ramírez-Vélez R. (2019). Handgrip strength attenuates the adverse effects of overweight on cardiometabolic risk factors among collegiate students but not in individuals with higher fat levels. Sci. Rep..

[18] Stewart A., Marfell-Jones M. (2011). International standards for anthropometric assessment. International Society for the Advancement of Kinanthropometry.

[19] Ramírez-Vélez R., Correa-Bautista J.E., Sanders-Tordecilla A., Ojeda-Pardo M.L., Cobo-Mejía E.A., Castellanos-Vega R.d.P., García-Hermoso A., González-Jiménez E., Schmidt-Riovalle J., González-Ruíz K. (2017). Percentage of body fat and fat mass index as a screening tool for metabolic syndrome prediction in Colombian university students. Nutrients.

[20] Rodríguez-Rodríguez F., Cristi-Montero C., González-Ruíz K., Correa-Bautista J.E., Ramírez-Vélez R. (2016). Bioelectrical impedance vector analysis and muscular fitness in healthy men. Nutrients.

[21] Ramírez-Vélez R., Correa-Bautista J.E., Carrillo H.A., González-Jiménez E., Schmidt-Riovalle J., Correa-Rodríguez M., García-Hermoso A., González-Ruíz K. (2018). Tri-ponderal mass index vs. Fat mass/height3 as a screening tool for metabolic syndrome prediction in colombian children and young people. Nutrients.

[22] Friedewald W.T., Levy R.I., Fredrickson D.S. (1972). Estimation of the concentration of low-density lipoprotein cholesterol in plasma, without use of the preparative ultracentrifuge. Clin. Chem..

[23] Mancia G., Fagard R., Narkiewicz K., Redón J., Zanchetti A., Böhm M., Christiaens T., Cifkova R., De Backer G., Dominiczak A. (2013). Task force members 2013 ESH/ESC guidelines for the management of arterial hypertension. J. Hypertens..

[24] Deboer M.D., Gurka M.J. (2017). Clinical utility of metabolic syndrome severity scores: Considerations for practitioners. Diabetes Metab. Syndr. Obes. Targets Ther..

[25] Schröder H., Fitó M., Estruch R., Martínez-González M.A., Corella D., Salas-Salvadó J., Lamuela-Raventós R., Ros E., Salaverría I., Fiol M. (2011). A short screener is valid for assessing mediterranean diet adherence among older Spanish men and women. J. Nutr..

[26] Estruch R., Ros E., Salas-Salvadó J., Covas M.-I., Corella D., Arós F., Gómez-Gracia E., Ruiz-Gutiérrez V., Fiol M., Lapetra J. (2013). Primary prevention of cardiovascular disease with a mediterranean diet. N. Engl. J. Med..

[27] Saydah S., Bullard K.M.K., Imperatore G., Geiss L., Gregg E.W. (2013). Cardiometabolic risk factors among US adolescents and young adults and risk of early mortality. Pediatrics.

[28] Kim M.K., Han K., Kwon H.S., Song K.H., Yim H.W., Lee W.C., Park Y.M. (2014). Normal weight obesity in Korean adults. Clin. Endocrinol..

[29] Zhang M., Schumann M., Huang T., Törmäkangas T., Cheng S. (2018). Normal weight obesity and physical fitness in Chinese university students: An overlooked association. BMC Public Health.

[30] Suliga E., Ciesla E., Głuszek-Osuch M., Rogula T., Głuszek S., Kozieł D. (2019). The usefulness of anthropometric indices to identify the risk of metabolic syndrome. Nutrients.

[31] Amirabdollahian F., Haghighatdoost F. (2018). Anthropometric indicators of adiposity related to body weight and body shape as cardiometabolic risk predictors in british young adults: Superiority of waist-to-height ratio. J. Obes..

[32] Yang H., Xin Z., Feng J.P., Yang J.K. (2017). Waist-to-height ratio is better than body mass index and waist circumference as a screening criterion for metabolic syndrome in Han Chinese adults. Medicine.

[33] Ashwell M., Gibson S. (2016). Waist-to-height ratio as an indicator of early health risk: Simpler and more predictive than using a matrix based on BMI and waist circumference. BMJ Open.

[34] St-Onge M.P., Janssen I., Heymsfield S.B. (2004). Metabolic syndrome in normal-weight Americans: New definition of the metabolically obese, normal-weight individual. Diabetes Care.

[35] Ramírez-Vélez R., Garcia-Hermoso A., Prieto-Benavides D.H., Correa-Bautista J.E., Quino-Ávila A.C., Rubio-Barreto C.M., González-Ruíz K., Carrillo H.A., Correa-Rodríguez M., González-Jiménez E. (2019). Muscle mass to visceral fat ratio is an important predictor of the metabolic syndrome in college students. Br. J. Nutr..

[36] Garcia-Hermoso A., Correa-Bautista J.E., Izquierdo M., Tordecilla-Sanders A., Prieto-Benavides D., Sandoval-Cuellar C., González-Ruíz K., Ramírez-Vélez R. (2019). Ideal cardiovascular health, handgrip strength, and muscle mass among college students: The fuprecol adults study. J. Strength Cond. Res..

[37] Ramírez-Vélez R., Correa-Rodríguez M., Izquierdo M., Schmidt-Riovalle J., González-Jiménez E. (2019). Muscle fitness to visceral fat ratio, metabolic syndrome and ideal cardiovascular health metrics. Nutrients.

[38] Wang Q., Zheng D., Liu J., Fang L., Li Q. (2019). Skeletal muscle mass to visceral fat area ratio is an important determinant associated with type 2 diabetes and metabolic syndrome. Diabetes Metab. Syndr. Obes. Targets Ther..

[39] Moy F.M., Loh D.A. (2015). Cardiometabolic risks profile of normal weight obese and multi-ethnic women in a developing country. Maturitas.

[40] Gómez-Ambrosi J., Silva C., Galofré J.C., Escalada J., Santos S., Millán D., Vila N., Ibãez P., Gil M.J., Valentí V. (2012). Body mass index classification misses subjects with increased cardiometabolic risk factors related to elevated adiposity. Int. J. Obes..

[41] Kobayashi Y., Niu K., Guan L., Momma H., Guo H., Cui Y., Nagatomi R. (2012). Oral health behavior and metabolic syndrome and its components in adults. J. Dent. Res..

[42] Shimpi N., Pathak R., Acharya A. (2019). Interdisciplinary care model: Metabolic syndrome and oral health. Integration of Medical and Dental Care and Patient Data.

